# HIV Incidence and Predictors Associated with Retention in a Cohort of Men Who have Sex with Men in Yangzhou, Jiangsu Province, China

**DOI:** 10.1371/journal.pone.0052731

**Published:** 2012-12-28

**Authors:** Zhihang Peng, Haitao Yang, Jessie Norris, Xin Chen, Xiping Huan, Rongbin Yu, Ning Wang, Hongbing Shen, Feng Chen

**Affiliations:** 1 Department of Epidemiology and Biostatistics, School of Public Health, Nanjing Medical University, Nanjing, Jiangsu, China; 2 Jiangsu Province Center for Disease Control and Prevention, Nanjing, Jiangsu, China; 3 National Center for AIDS/STD Control and Prevention, Chinese Center for Disease Control and Prevention, Beijing, China; Public Health Agency of Barcelona, Spain

## Abstract

**Background:**

The study was to investigate the incidence of HIV-1 and related factors, as well as predictors associated with retention in a cohort study among men who have sex with men (MSM) in Yangzhou, Jiangsu Province, China. A carefully designed 12-month prospective cohort study was conducted.

**Methodology/Principal Findings:**

A total of 278 sero-negative MSM were recruited and followed up for 12 months starting from May, 2008. Participants were tested for HIV-1 at baseline, 6-month, and 12-month follow-up visits. Questionnaire interviews were conducted to collect information. The retention rate and HIV incidence were analyzed as functions of demographic and behavioral variables. Risk factors were identified by estimating the relative risks (RR) and respective 95% confidence intervals (CI) using a Poisson regression model, univariate and multivariate analyses and risk factors analyses. 71 (25.5%) and 45 (16.2%) of the 278 participants were retained at the 6-month and 12-month follow-up visits respectively. The incidence rates of HIV-1 were 5.65 and 6.67 per 100 person years (PY) respectively. Both having received condoms and having received lubricant were negatively associated with HIV sero-conversion at the 12 months’ follow-up. Predictors associated with 12-month retention rate include Yangzhou residency (RR = 0.471, 95%CI: 0.275∼0.807, P = 0.006), having received condoms (RR = 0.065, 95%CI: 0.007∼0.572, P = 0.014), and having received VCTs (RR = 0.093, 95%CI: 0.010∼0.818, P = 0.032).

**Conclusions/Significance:**

The incidence of HIV-1 among MSM in Yangzhou is relatively high and effective interventions are needed urgently. More attention should be focused on maintaining a higher retention rate.

## Introduction

HIV/AIDS was first identified among men who have sex with men (MSM) in the United States in the early 1980s [1]. As time passed, MSM populations gradually manifested a high burden of HIV in many countries around the world. In the Western world, the rate of new HIV infections among MSM continues increasing; in the United States in 2006, 49% of new HIV/AIDS diagnoses were among men who have sex with men (MSM). HIV infections in MSM are estimated to be increasing at roughly 8% per year since 2001. [2], [3] In the developing world, epidemiologic studies have now established the widespread existence of HIV in MSM populations in Africa, China [4] and Russia [5]. High and increasing HIV prevalence rates have also been reported from South and Southeast Asia, the Caribbean and Latin America. [6] [7], [8] Rapid increases in reports of new HIV diagnoses among MSM have also been seen in recent years, particularly in East Asia [2]. In Asia, studies in Bangkok, Thailand, show that HIV prevalence among MSM increased from 17.3% in 2003 to 28.3% in 2005 [9], [10].

In mainland China, the number of MSM has skyrocketed because of economic development and ideological emancipation. The MSM population is currently estimated at between 2 and 8 million [11], [12], with 47 000 of them estimated to be living with HIV/AIDS. [13] In 2005, 17.3% of all estimated HIV cases could be attributed to MSM behaviors [14]. In 2007, 12.2% of the 50,000 estimated new infections could be attributed to MSM, third behind both Injecting Drug Users (IDUs) and sex workers (SWs) and their clients [15], which is much higher than the 2005 estimate (7.5%). [16], HIV prevalence among MSM has increased rapidly in recent years in many different parts of China. In Beijing, the rates went from 0.4 percent to 4.6 percent between 2004 and 2005, and afterwards increased to 5.8 percent in 2006, indicating a clear upward trend. [17] In Shandong Province, the rate rose from 0.05% in 2007 to 3.1% in 2008 [18].

MSM are highly vulnerable to HIV infection due to unprotected anal intercourse and multiple sexual partners. [19], [20] Self-discrimination, or discrimination and censure from society that makes a group feel shameful, guilty or inferior, is remarkably prevalent among MSM because of traditional Chinese culture, which has had a critical impact on their individual behavior, psychological health status and prevalence of AIDS. [21], [22] MSM tend to disguise their sexual orientation by marrying to female spouses, which makes them hard-to-reach population [23] and potentially increases the rate of HIV infection in the general population. More focus ought to be given to MSM populations in China and the role of their behaviors in HIV/AIDS transmission [12].

Most of the aforementioned studies were cross-sectional and few reported incidence data. However, reporting HIV incidence data is crucial in that it can describe the rate at which HIV is spreading in a target population, foretell future trends of HIV prevalence, and quantify numbers of new infections appearing in given a period of time. Aside from improved HIV-related knowledge, reduction of HIV incidence is currently the strongest measure to evaluate whether HIV prevention services are effective in controlling the epidemic, which allows for better allocation of public resources in the future. [24] So far, few cohort studies have been conducted in China. In Beijing, sero-incidence was 3.37/100 person-years (95% CI = 1.66–5.08) in a 12-month cohort study of 525 HIV-negative MSM in 2008, [25] which was higher than that of another similar study in Beijing in 2007 (2.6/100 PY). [26] In Nanjing, the rate was 5.12/100 PY according to a 6-month cohort of 397 MSM in 2007. [24] In addition to the studies above, a 12-month cohort study also took place in Shenyang, which revealed an incidence of 5.4/100 PY for HIV among 218 MSM between 2006 and 2007. [27] However, the HIV incidence of MSM in smaller cities around China remains largely unknown. Since homosexuality is influenced profoundly by the economy, culture, custom and values of the society, characteristics such as the size of a city may lead to diversity in ideas and behaviors among the MSM population. Our interventions should be tailored according to the characteristics of mid-sized cities, then extend to other middle and smaller cities.

Yangzhou is a hub city of both Shanghai’s economic circle and Nanjing’s metropolitan circle in Jiangsu Province. According to a cross-sectional survey, the HIV prevalence of MSM in Yangzhou was 8.03% in 2007, which was higher than that of Nanjing (4.73%). [19] Due to the small size of this city, it may be easily overlooked and infection levels insufficiently monitored until a sudden outbreak occurs. It is necessary for us to profoundly investigate the possible risk behaviors of MSM in Yangzhou so that effective measures can be taken to reduce their chances of HIV infection.

Our study reports on HIV incidence obtained from a cohort of 278 HIV-negative MSM in Yangzhou, China. We aim to identify predictors of HIV infection and to maintain a high cohort retention rate through analyzing the factors associated with them. We hope our study will be a boon for future studies, designers of innovative intervention measures and government representatives responsible for resource allocation in the fight against HIV transmission among MSM in Yangzhou, China.

## Materials and Methods

### Study Design and Study Population

This prospective cohort study was conducted among MSM in Yangzhou, China. A baseline screening survey was conducted starting in May, 2008. Study participants were recruited online using a snowball sampling method. Eligible participants of the baseline study were males who (1) had a long-term residence in Yangzhou city lasting more than 3 months; (2) had previously engaged in anal or oral sex with a man in the past 12 months; (3) were at least 18 years old.

Interviews and voluntary counseling and testing (VCT) procedures were conducted at an HIV clinic conveniently located at the Yangzhou Center for Disease Control and Prevention. Privacy was ensured. Participants were informed about the results of the rapid HIV test and posttest counseling was provided by a medical doctor. Those yielding a positive rapid test result were asked to return in person in 2 weeks to receive confirmatory testing results. Those with confirmed HIV-positive status received additional posttest counseling and referrals to relevant free services.

Verbal and written informed consents were obtained from respondents before the anonymous, face-to-face interview commenced. In order to acquire a high retention rate, some steps were arranged in advance. All participants who received HIV antibody testing, regardless of whether they succeeded in recruiting other participants, were given a box of free condoms and lubricant. An amount of 30 RMB (4.63 USD) was also added to their mobile phone account as an incentive. Besides, well trained linkmen contacted with the participants at regular intervals to master their latest contact information and reminding calls were made before follow-up day to make sure that they will show up on time or just make another date if they have no time to spare.

### Data Collection and Laboratory Tests

Questionnaire-based interviews were conducted on a one-on-one basis in a separate and private room of the district clinic. The questions were drawn from the core questionnaire for the National MSM Comprehensive Preventative and Treatment Experimental Unit Project, with some variables added that were specific to Jiangsu Province. Sociodemographic characteristics, Knowledge, Attitudes, and Practice (KAP) about AIDS, sexual behaviors, and other such variables were also addressed. Trained peer field workers conducted the interviews at baseline, 6-month follow-up and 12-month follow-up visits, before participants received their VCT services.

Blood specimens were collected. The HIV antibody was tested by a rapid blood HIV antibody test (ELISA; Diagnostic Kit for Antibody to Human Immunodeficiency Virus, Acon Biotech Co., Ltd., China). Positive screening results were retested by another rapid test (Serodia-HIV; Fujirebio Inc., Japan), and Western Blot assay was used as the confirmatory test (Genelabs Diagnostics Pte Ltd., Singapore). Syphilis screening was performed using the Rapid Plasma Reagin Test (RPR; Beijing WanTai, Biologic Pharmacy Enterprise Co. Ltd). Positively screened results were confirmed using the Treponema Pallidum Particle Agglutination Test (TPPA; Livzon Pharmaceutical Group Inc.). Counseling and clinical treatment were offered to all cases with confirmed syphilis testing results.

### Statistic Analysis

Questionnaire-based data and biological testing results were recorded, double checked, and compared with EpiData software (Epi Data for Windows; The Epi Data Association Odense, Denmark). After corrections, data were then converted and analyzed using a statistical analysis system (SAS 9.1 for Windows; SAS Institute Inc., NC). Statistical significance was defined by P value<0.05.

The HIV incidence rate was calculated using number of seroconversions within the follow-up period as the numerator and the cohort’s total number of person-years (PY) of exposure to the risk of HIV transmission as the denominator. The Poisson method was used to estimate the 95% confidence interval (CI) of the incidence. A multiple logistic regression model was constructed to select independent factors for the retention rate.

## Results

### Sociodemographic Characteristics and Sexual Behaviors of Study Participants at Baseline

The 278 participants had a median age of 32.0±10.6 years. Approximately half (51.0%) were single, 41.3% were married, 2.7% were cohabitating, and 5.0% were divorced or widowed. Most participants (99.3%) belonged to Han ethnic group. Non-Yangzhou residents accounted for 60.0%, among whom 34.7% lived in Jiangsu Province while 25.3% lived in other provinces. A total of 28.3% had completed junior college or higher levels of education. Most (37.7%) had monthly incomes between 1001 and 2000 yuan. Another sizeable proportion (21.0%) had monthly incomes of 2001–3000 yuan. Participants who identified themselves as exclusively homosexual or bisexual accounted for 38.3% and 55.7%, respectively. The median number of sexual partners was 9.2. Most (82.7%) participants had had anal sex with a male partner in the last 6 months. In homosexual encounters over the last 6 months, 22 (8.9%) participants had never used condoms, and 52.0% of them had used condoms every time. 153 (61.7%) participants had anal sex with regular partners in the last 6 months, among whom 49.0% invariably used condoms. 186 (75.0%) participants had had casual sex partners, among whom 64.5% always used condoms in the last 6 months. 7.7% of the 248 participants had paid for sex, and 14.1% had sold sex. About half (48.7%) of them had had sex with a female in the last 6 months, among whom only 21.2% used one every time. In the past year, 48.3% had ever received condoms and 23.3% had ever received lubricant. 36.0% of participants had ever taken part in peer-education programs and 51.7% had previously received VCTs. (Table 1).

**Table 1:** Sociodemographic Characteristics of the 300 study participants in the cohort of men who have sex with men in Yangzhou, China.

Variables	n	%
Overall N	300	100
Age (median, yrs)	32.0±10.6	
Marital Status		
Single	153	51
Married	124	41.3
Cohabited	8	2.7
Divorced/widowed	15	5
Ethnicity		
Han	298	99.3
Minority	2	0.7
Registered permanent residence		
Local city	120	40
Other places in local province	104	34.7
Other provinces	76	25.3
Sex Orientation		
Homosexual	115	38.3
Heterosexual	8	2.7
Bisexual	167	55.7
Indeterminate	10	3.3
Education level		
Junior college and below	215	71.7
Above junior college	85	28.3
Monthly income (yuan)		
≤1000	68	22.7
>1000	232	77.3
Had anal sex with male partners in the last 6 months	248	82.7
In the last 6 months’ homosexual encounters		
Have never used condoms	22	8.9
Used condoms sometimes	97	39.1
Used condoms every time	129	52
Sex with regular partners in the last 6 months		
Stuck to using condoms	75	49
Sex with casual partners in the last 6 months		
Consistently used condoms	120	64.5
Have bought sex in the last 6 months	19	7.7
Have sold sex in the last 6 months	35	14.1
Sex with female in the last 6 months		
Have never used condoms	72	49.3
Used condoms sometimes	43	29.5
Used condoms every time	31	21.2

### HIV Incidence at the 6-month Follow-up Visit and 12-month Follow-up Visit

Among the 71 participants who appeared at the 6-month’s visit, 2 were HIV positive at the first screening, yielding an incidence rate of 5.6 per 100 PY. 45 participants appeared at the 12 months’ visit and 3 of them had sero-converted, indicating an incidence rate of 6.67 per 100 PY. No predictor was found associated with HIV sero-conversion when using chi-test and logistic regression analyses. Risk factor analyses showed that both condoms and lubricant provision played an important role in preventing sero-conversion.

### Factors Associated with Retention Rate

According to logistic analyses, those who were Yangzhou permanent residents (RR = 0.471, 95%CI: 0.275∼0.807, P = 0.006), had received condoms (RR = 0.065, 95%CI: 0.007∼0.572, P = 0.014) and had received VCTs (RR = 0.093, 95%CI: 0.010∼0.818, P = 0.032) were more likely to appear at the 12-month follow-up visit. (Table 2∼3).

**Table 2:** Factors Associated With 6 months’ Retention Rate in a Cohort Study among MSM in Yangzhou, China, Using logistic analyses.

Factors	N	Retention Rate %(n)	RR(95%CI)	*P*
Yangzhou permanent residents				
Yes	108	39.8(43)	1.000	
No	170	16.5(28)	2.976(1.779, 4.975)	<0.001
Have received condoms				
Yes	136	50.7(69)	1.000	
No	142	1.4(2)	10.420(1.692, 62.500)	0.011
Have received lubricants				
Yes	68	94.1(64)	1.000	
No	210	3.3(7)	3.497(1.050, 11.628)	0.041
Have received VCTs				
Yes	146	46.6(68)	1.000	
No	132	2.3(3)	14.925(3.861, 58.824)	<0.001

**Table 3:** Factors Associated With 12 months’ Retention Rate in a Cohort Study among MSM in Yangzhou, China, Using logistic analyses.

Factors	N	Retention Rate %(n)	RR(95%CI)	*P*
Yangzhou permanent residents				
Yes	108	28.7(31)	1.000	
No	170	8.2(14)	2.123(1.239, 3.636)	0.006
Have received condoms				
Yes	136	32.4(44)	1.000	
No	142	0.7(1)	15.385(1.748, 142.857)	0.014
Have received VCTs				
Yes	146	30.1(44)	1.000	
No	132	0.8(1)	10.753(1.223, 100.000)	0.032

## Discussion

Our findings show that HIV incidence (5.56 and 6.67/100PY) in Yangzhou is alarmingly high and the figure dovetails with the sharply increasing HIV prevalence (8.03% in 2007) among MSM in Yangzhou. [19] Beyond our expectations for a smaller city, its HIV incidence is even higher than those of large metropolises such as Nanjing (5.12/100PY in 2007), Beijing (3.37/100PY in 2008 and 2.6/100PY in 2007) and Shenyang (5.4/100PY in 2006 to 2007). [24], [25], [26], [27] One major reason for this outcome could be that Yangzhou is an important city located in a developed region and adjacent to some larger metropolises; MSM from nearby cities may be more prone to looking for partners there, away from their own residences, to avoid being discovered by acquaintances. [28] In addition, Yangzhou is a beautiful city attracting many tourists, which may facilitate a population flow. As compared with these metropolises, it can be inferred that Yangzhou is not so cosmopolitan that the knowledge and perceived risks of HIV infection among its citizens, including the MSM population, are sufficient. Besides, a discriminatory and deprecatory attitude toward homosexuality here may be more widespread and deep-rooted. The high incidence rate indicates that the related high risk behaviors need to be identified, and comprehensive interventions targeted for MSM are urgently required not only in large cities but also in smaller cities like Yangzhou.

In our study, MSM who had been provided with condoms or lubricant showed a relatively lower incidence rate and a higher retention rate, which laterally indicates the effects of condoms and lubricant in preventing HIV transmission. Through receiving condoms and lubricant services, this population may have been subconsciously influenced by protective awareness and at the same time given the means to protect itself. To some extent, they may have realized the significance of studying MSM as a specific population and as a result remained with the investigation in its later phases to show their support.

Having received VCTs (voluntary counseling and testing) may present a similar effect. Those who received the intervention showed a higher retention rate, as expected. All this demonstrates the necessity of promoting AIDS-related services more frequently. It is hoped that more knowledge will spur the target population into action. In smaller cities like Yangzhou, there always fewer HIV testing institutions or non-government organizations offering VCTs, and fewer physicians who frequently test for syphilis and regularly recommend tests for HIV. This emphasizes the need to train physicians in providing accurate STD screening, standard care, and treatment for MSM in Yangzhou and other smaller cities to reduce the risk for HIV acquisition or transmission. In view of the fact that most participants enrolled for this study were recruited through the Internet and given the wide Internet coverage rate in Yangzhou, we can see that the Internet played an important role in looking for partners. We should take advantage of the Internet to make it a useful approach for disseminating information in Yangzhou.

Actually, since 2005, the Chinese government has strengthened its interventions for MSM populations, which include promoting condom use, counseling and testing, peer education, and STI services. Third quarter 2007 statistics reported that the interventions have reached 88,082 MSM, covering about 8.2% of the MSM population. [29] Meanwhile, studies on HIV and MSM in China have mushroomed in recent years.

Some strategies have been also issued since 2004 to promote 100% condom use among MSM. The establishment of training workshops, condom distributions and social marketing programs were included. [30] It is prudent for many research groups to distribute condoms and lubricant to the study populations; however, condom use levels still remain far from acceptable. A study of 14 cities in China showed that the proportion of people in the last month who consistently used condoms each time with male partners was only 39.2%, and only 65.2% of participants used condoms with male partners in their last sexual encounter. [31] Only 32.5% of 1,389 participants had always used condoms during anal intercourse in a study that covered six cities in China. [32] In our first study, more than half of the participants had never received free condoms or lubricant or peer education, and nearly half had never received AIDS voluntary counseling and testing services. Thus, condom promotion shoulders heavy responsibilities. There is a perpetual need for measures aiming to increase knowledge about condoms, particularly measures that utilize education about proper use, condom social marketing and quality assurance [33].

In addition, most MSM have one or more regular sex partners, and the existence of casual ones among their male sex partners in the last 6 months indicates that some MSM have a more active sexual life, which adds to the risk of HIV infection. According to a study [34] that combined the Theory of Planned Behavior and the Norm-Activation Theory, HIV-positive MSM are more likely to engage in unprotected anal intercourse (UAI) in the context of casual sex encounters than in steady sexual relationships. However, some other studies have shown a higher rate of UAI with regular compared to casual partners. [35], [36] As an issue influenced by multiple factors, relationships between UAI and regular or casual partners deserve further discussion.

In our study, 44% of the participants were married or cohabitating, 55.7% and 2.7% were self-identified bisexual or heterosexual, respectively, and almost 50% of those who had had sex with females reported never using condoms in the last 6 months. This serves as a warning sign on the bridge effect of AIDS transmission between a high-risk population and the general population. Some measures should be taken to encourage MSM to assume more responsibility for their families by using condoms more frequently inside the home, for there are currently no good measures to protect female partners of MSM from being infected with HIV.

In this prospective cohort study, 25.5% and 16.2% of the participants successfully completed the 6-month and 12-month cohort, respectively, which were rather low rates compared with others. The HIV Network for Prevention Trials Vaccine Preparedness Study has successfully followed up with 88% of 3257 MSM participants in 6 US cities over an 18-month period. [37] In China, this rate was slightly lower. For example, two studies in Nanjing reported 71.84% [38] and 72%, [24] respectively; two studies conducted in Beijing had rates of 87% [25] and 86.2% [23].

Those who were not Yangzhou permanent residents were more likely to be lost-to-follow-up at the 6-month and 12-month visits. A plausible reason is that it was less convenient for them to be followed up with compared to those who had settled down in Yangzhou. They also may have had less access to local preventive services. This also implies that migrant populations may play a role in HIV infection among MSM in Yangzhou, and investigations of a deeper character should be conducted among this subpopulation.

In addition to the above-mentioned residency aspect, some other reasons can be inferred. On the one hand, most of them had below a junior college (71.7%) level of education, which may contribute to poor knowledge of AIDS and a lack of awareness or disregard for the dangers this disease may cause. Participants who were concerned about the progress of AIDS research and related health problems showed better compliance with our study. Since this would apparently reduce the loss-to-follow-up rate, some simple mobilization campaigns emphasizing the significance of the study should be added to improve MSM’s sense of ownership in the research. In addition, homosexuality is traditionally stigmatized and unaccepted in China, so participants undergo physical and mental burdens that include fear of disclosure of their sexual identities, which may have a great negative impact on their family and personal lives. For this study, participants were all recruited from the Internet and there was little basis for trust between participants and researchers, so our only option in this initial foray into the community was to make a more practical guarantee of establishing a good reputation and building trust. Participants should also be more respected during the whole study. [39] Last but not least, although our study shows that most participants had a stable income, they may still be too busy with their lives and work to pay attention to their health. If it were not for these factors, perhaps the payoff in follow-up rate would have been more generous.

This study has a number of limitations. Firstly, retention rates were so low that it may have indicated a HIV sero-conversion rate higher than in similar studies because no obvious evidence has been showed the balance between follow-ups and the lost. Secondly, the sample size was so small that the rates may have deviated slightly and some potential important factors may be left out. The Internet-only method of recruiting may have led to a selection bias. Third, like other studies, condom use was self-reported and this added to uncertainty about the accuracy of results. Fourthly, only major high-risk factors were investigated here; other behavioral and psychological details that may change HIV incidence rates were not examined. Lastly, there is not enough evidence that our study results could be applied to other regions of China.

This study was carefully designed, performed, and monitored. It demonstrates the urgency of the high HIV epidemic in Yangzhou, China. Although it is not possible to extrapolate these results to all MSM, provides some potentially useful information for designing future programs aiming at middle and small cities. Along with more incidence studies, we hope our study will be a helpful reference for the fight against HIV among MSM in China.
